# Role of CD1d and iNKT cells in regulating intestinal inflammation

**DOI:** 10.3389/fimmu.2023.1343718

**Published:** 2024-01-11

**Authors:** Sung Won Lee, Hyun Jung Park, Luc Van Kaer, Seokmann Hong

**Affiliations:** ^1^ Department of Biomedical Laboratory Science, College of Health and Biomedical Services, Sangji University, Wonju, Republic of Korea; ^2^ Department of Integrative Bioscience and Biotechnology, Institute of Anticancer Medicine Development, Sejong University, Seoul, Republic of Korea; ^3^ Department of Pathology, Microbiology and Immunology, Vanderbilt University School of Medicine, Nashville, TN, United States

**Keywords:** invariant NKT cells, inflammatory bowel diseases, CD1d, glycolipid antigens, commensal bacteria, short-chain fatty acids

## Abstract

Invariant natural killer T (iNKT) cells, a subset of unconventional T cells that recognize glycolipid antigens in a CD1d-dependent manner, are crucial in regulating diverse immune responses such as autoimmunity. By engaging with CD1d-expressing non-immune cells (such as intestinal epithelial cells and enterochromaffin cells) and immune cells (such as type 3 innate lymphoid cells, B cells, monocytes and macrophages), iNKT cells contribute to the maintenance of immune homeostasis in the intestine. In this review, we discuss the impact of iNKT cells and CD1d in the regulation of intestinal inflammation, examining both cellular and molecular factors with the potential to influence the functions of iNKT cells in inflammatory bowel diseases such as Crohn’s disease and ulcerative colitis.

## 1 Introduction

Inflammatory bowel disease (IBD) is a chronic inflammatory disorder of the intestine stemming from an imbalanced immune response to gut microflora rather than infectious causes. IBD manifests primarily as two forms: Crohn’s disease (CD) and ulcerative colitis (UC). Whereas CD affects the entire digestive tract, UC primarily involves inflammation in the colon and rectum (1, 2). Environmental factors such as a high-fat diet (HFD) and hormonal substances contribute to the increasing incidence of IBD in developed countries (1, 2). Although various immune cells have been implicated in the pathogenesis of IBD, we focus here on natural killer T (NKT) cells, a subset of innate-like T cells that play a crucial role in maintaining intestinal integrity. Unlike conventional T cells, NKT cells co-expressing NK cell receptors and T cell receptors (TCRs) respond to glycolipid antigens (Ags) presented by the major histocompatibility complex class I-related protein CD1d. CD1d-restricted NKT cells mainly consist of type I invariant NKT (iNKT) and type II NKT cells that react to glycolipids, α-galactosylceramide (α-GalCer) and sulfatide, respectively (3–5). Human iNKT cells include mainly CD4^+^, CD8^+^, and CD4^-^CD8^-^ double-negative (DN) subsets, whereas murine iNKT cells consist of CD4^+^ and DN subsets. Moreover, peripheral blood iNKT cells occupy 0.1-0.2% and 1-2% of T cells in humans and mice, respectively. Type I iNKT cells are less abundant than type II NKT cells in human liver, but opposite relative frequencies are observed in mouse liver (6, 7).

NKT cells can produce various cytokines including IFN-γ, IL-4, IL-10, IL-13, and IL-17 (8–10). Moreover, iNKT cells are functionally categorized based on their preferential expression of transcription factors: T-bet for iNKT1, GATA3 and PLZF for iNKT2, RORγt for iNKT17, and E4BP4 for iNKT10 cells (11, 12). iNKT1 cells are predominant in the spleen and the liver, whereas iNKT2 cells predominantly localize to mesenteric lymph nodes (mLNs) (5, 11). IL-10-expressing iNKT10 cells are predominant in the visceral adipose tissue (VAT) as well as in the colonic lamina propria (LP) (13, 14). Whereas B6 mice contain a prominent population of iNKT1 cells in the thymus, Balb/c mice contain higher frequencies of iNKT2 cells in the thymus and mLNs (5). It was recently reported that non-immune cells (i.e., intestinal epithelial cells (IECs) and serotonin-secreting enterochromaffin cells) constitutively express CD1d molecules that can present glycolipid Ags (15–17). Although the canonical role of CD1d in lipid Ag presentation is well-characterized in immune cells such as dendritic cells (DCs), increasing evidence also shows non-canonical CD1d functions in CD1d-expressing mononuclear cells, including type 3 innate lymphoid cells (ILC3s) and IECs. Besides their capacity to present lipid Ag, CD1d molecules can intrinsically signal cells to become activated and produce cytokines (17–19). Emerging evidence indicates that intrinsic CD1d signaling in intestinal cells (i.e., tuft cells, Paneth cells, enterocytes, goblet cells, and neuroendocrine cells) has clinical implications in IBD. The quantity and phenotype of iNKT cells are altered in IBD patients compared with healthy individuals (13, 20). Moreover, since iNKT cells can produce pro- and anti-inflammatory cytokines, iNKT cell-elicited immune responses may be protective or harmful. This mini-review will focus on the immunomodulatory roles of CD1d and iNKT cells in two primary forms of IBD, CD and UC.

## 2 NKT cells in IBD

In CD, exaggerated T helper (Th)1 and Th17 immune responses characterize pathogenic lesions across the gastrointestinal tract (1, 2). CD patients exhibit a significant reduction in iNKT cell numbers in peripheral blood and intestinal tissues (20). Likewise, the acute mouse IBD model induced with dextran sulfate sodium (DSS) is characterized by increased Th1 and Th17 immune responses (21). iNKT cell-deficient mice display increased colitis severity in experimental models, indicating a regulatory role for colonic iNKT cells, particularly those producing anti-inflammatory cytokines like IL-9 and IL-10 (13, 22). Recently, we have demonstrated that IL-4- and IL-9-producing iNKT cells are significantly increased in the mLNs during IFN-γ-mediated intestinal inflammation after DSS treatment (23). Furthermore, consistent with our report, the prevalence of anti-inflammatory iNKT10 cells is elevated in CD patients compared to healthy individuals, suggesting the existence of regulatory pathways that compensate for the production of harmful pro-inflammatory cytokines in the intestine of CD patients (13).

In contrast to CD patients, IL-13-producing cell populations in the LP of UC patients contain CD1d-restricted type II NKT cells but not iNKT cells (24). Overexpression of CD1d in a type II NKT TCR transgenic (Tg) mouse model (CD1d^Tg^/24αβ^Tg^) forces the negative selection of type II NKT cells, consequently reducing the frequency of type II NKT cells (25). Thus, type II NKT cell-deficient CD1d^Tg^/24αβ^Tg^ mice spontaneously develop colitis, indicating that type II NKT cells play essential roles in maintaining intestinal homeostasis (25). However, LP mononuclear cells (including type II NKT cells) from UC patients express high levels of CD161 and IL-13Rα and produce high amounts of IL-13. Upon stimulation with sulfatide glycolipids, these cells exhibit augmented cytotoxic activity against IECs (26). In addition, a methionine-choline-deficient (MCD) diet lowers the type II NKT population in both the LP and mLNs, ultimately suppressing DSS-induced colitis (27). These findings indicate that type II NKT cells can be either protective or pathogenic in UC. Since IFN-γ produced during intestinal inflammation contributes to the relative distribution of type I and II NKT cells (28, 29), the balance of these subsets plays a significant role in regulating IBD.

## 3 Effect of glycolipid-stimulated iNKT cells on IBD

Exposure to mucosa-associated microbiota drives pro-inflammatory activation of colonic iNKT cells (mostly CD161^+^ cells) from IBD patients through TCR-dependent and -independent mechanisms, consequently breaking the epithelial barrier integrity (30). The presentation of commensal-derived glycolipid Ags and exogenous glycolipids by CD11c^+^ cells, such as DCs, controls the homeostasis and activation of intestinal iNKT cells (31). Furthermore, Wingender et al. demonstrated that intragastric injection of *Sphingomonas yanoikuyae* containing glycolipid Ags activates intestinal iNKT cells with a hyporesponsive phenotype and increases the relative frequency of Vβ7^+^ iNKT cells in germ-free mice (32). Repeated intraperitoneal (i.p.) injection of either α-GalCer or its more potent analog (7DW8-5) (33, 34) into DSS-treated mice effectively prevents colitis development. Moreover, repeated i.p. injection of α-GalCer inhibits the development of cholangitis complicated by colitis in outbred CD-1 mice by reducing Th1-dominant responses (35). Since repeated i.p. or intravenous (i.v.) injection of α-GalCer increases iNKT10 differentiation (10, 12), it will be worthwhile to investigate whether resolution of colitis by repeated i.p. α-GalCer injection correlates with an increase in colonic iNKT10 cells. Furthermore, repeated α-GalCer challenge induces memory-like cMAF^+^ iNKT cells and IL-10-expressing adipose tissue iNKT1 cells, suggesting that iNKT10 cells may differentiate from iNKT1 cells (14). On the other hand, α-GalCer-like glycolipid produced by the commensal bacterium *Bacteroides fragilis* (*B. fragilis*) stimulates splenic iNKT cells to secrete high amounts of IL-10 in Balb/c mice enriched for iNKT2 cells. Upon stimulation with these glycolipids, most iNKT10 cells simultaneously secrete IL-13 but not IFN-γ (36). Fecal microbiota transplantation (enriched for *Lactobacillaceae* and *Bifidobacteriaceae*) suppresses DSS-induced colitis, which is associated with increased iNKT10 cells (37, 38). However, the origin and differentiation of colonic iNKT10 cells during colitis remains unclear. Oral administration of α-GalCer specifically up-regulates expression of TCR engagement markers (e.g., Nur77) in iNKT cells of the mLNs but not the spleen, liver, and thymus and its up-regulated expression selectively occurs in iNKT2 rather than iNKT1 populations in the mLNs (5), supporting the notion that optimal glycolipid administration frequency and route are important factors in treating colitis. Injection of OCH, a derivative of α-GalCer with Th2 selective activity, prevents DSS-induced colitis, which correlates with reduced Th1/Th2 cytokine ratios and increased IL-10 production (39). Treatment with an α-GalCer derivative containing polar functional groups (Bz amide) attenuates DSS-induced colitis, accompanied by expansion and activation of Th2- and Th17-biased iNKT cells rather than Th1-biased iNKT cells (40). Glycolipid Ags derived from the intestinal microbe *B. fragilis* (called GSL-Bf717 or BfaGCs) inhibit iNKT cell activation through competitive binding of their sphinganine branches with CD1d, resulting in protection against oxazolone (Oxa)-induced colitis, an experimental model mimicking UC (41–43). Additional studies have provided evidence that CD1d-dependent pathogenic iNKT cell activation by commensal-derived glycolipid Ags can be inhibited by competitive CD1d binding with globotriaosylceramide (44) and α-lactosylceramide (45) during iNKT cell-mediated Oxa-induced colitis.

## 4 Cell-intrinsic role of CD1d in IBD

CD1d expression on IECs is significantly decreased in both CD and UC patients compared with healthy controls, suggesting a unique role in controlling intestinal immune responses (46). Exposure of environmental oxazoles derived from either diet or microbes to IECs can play pathogenic roles in colonic inflammation by inhibiting IEC-mediated and CD1d-dependent IL-10 production and promote iNKT cell-derived IL-13 production (47). Lipid-mediated CD1d ligation triggers epithelial cell-derived endogenous IL-10 production, consequently decreasing epithelial permeability induced by IFN-γ (48). Furthermore, one study demonstrated that IECs produce high amounts of IL-10 via CD1d engagement-induced STAT3 activation, suppressing Oxa-induced iNKT cell-mediated colitis (17). These studies suggest that the cross-talk between iNKT cells and CD1d-expressing IECs activated by CD1d-intrinsic signaling controls intestinal homeostasis.

In addition to its effects on IECs, CD1d engagement on various other cell types can induce cell-intrinsic signaling. In mLN B cells, CD1d expression is associated with enhanced IL-10 production under chronic intestinal inflammatory conditions (49). Since IL-10-producing B cells (B10) suppress experimental arthritis in a CD1d-dependent manner (50), it will be interesting to investigate whether iNKT cells cross-talk with B10 cells to regulate colitis. Glycolipid-mediated ligation of CD1d on human monocytes directly triggers marked NFκB activation and excessive IL-12 production, indicating that CD1d-mediated signal transduction is required to induce optimal Th1 responses to glycolipid Ag stimulation (51). Macrophage-specific CD1d deletion alleviates colonic inflammation, highlighting CD1d-intrinsic activating effects on NFκB in macrophages (19). CD1d-deficient macrophages exhibit metabolic reprogramming into an inflammatory phenotype through CD36 internalization and lipid uptake. In addition, mice reconstituted with CD1d-deficient macrophages exhibit increased susceptibility to LPS-induced inflammation (52). Engagement of CD1d on ILC3s by NKT cells drives IL-22 secretion in the mLNs (18). In addition, CD1d-dependent engagement of enterochromaffin cells by iNKT cells triggers the former cells to release peripheral serotonin (5-HT), thereby regulating intestinal hemostasis (16). Finally, a recent study showed that intrinsic hepatocyte-specific CD1d signaling induced by tyrosine phosphorylation of the CD1d cytoplasmic tail protects against hepatocyte apoptosis in mice with non-alcoholic steatohepatitis induced by an HFD or MCD diet (53).

iNKT cells are thought to play protective or pathogenic roles in colitis through their activation via TCR- or cytokine-mediated signaling pathways. Furthermore, various CD1d-expressing cells (e.g., B cells, ILC3s, and macrophages) that can interact with iNKT cells also appear to play protective or pathogenic roles in colitis by producing cytokines (e.g., IL-22 and IL-10) via CD1d-intrinsic signaling. Thus, it will be important to explore how CD1d-intrinsic signaling modulates the function of NKT cells and whether such interactions contribute to colitis development.

## 5 Immune cells that engage in cross-talk with iNKT cells during IBD

### 5.1 Myeloid lineage cells: macrophages and neutrophils

CX3CR1^hi^ mononuclear phagocytes guide non-invasive *Salmonellae* to the mLNs where these organisms induce both pathogen-specific T cell responses and IgA antibodies (54). During naive CD4^+^ T cell transfer-mediated colitis, CD4^+^ T cells are in close contact with colonic CX3CR1^+^ phagocytes that present bacterial-derived Ags (55). Interestingly, embryonic CX3CR1^+^ macrophages are essential for the localization and proliferation of colonic iNKT cells (particularly iNKT17) in their local environment during early life (56). In this regard, it will be worthwhile to further investigate whether CX3CR1^+^ macrophages contribute to iNKT cell-mediated host defenses during enteropathogen-induced colitis. Compared with macrophage recruitment, the effect of iNKT cells on neutrophil recruitment remains unclear. For example, the expression of neutrophil-attracting chemokines (i.e., CXCL1, CXCL2, and CXCL3) decreases in the colon of CD1d^-/-^ mice, along with reduced pathogenic neutrophil infiltration (57). In contrast, more neutrophils are recruited to the colon in DSS-treated iNKT cell-deficient Jα18^-/-^ mice and these cells possess a pronounced anti-inflammatory phenotype (58). Since neutrophils are classified into two groups based on their functional differences (59), pro-inflammatory N1 (e.g., TNF-α-secreting) and anti-inflammatory N2 neutrophils (e.g., TGF-β-secreting), further studies are needed to clarify the role of iNKT cells on neutrophil polarization (N1 vs. N2) during colitis development.

### 5.2 Innate lymphoid cells: ILC2 and ILC3

Blockade of IL-25 signaling is an effective strategy to prevent intestinal inflammation in Oxa-induced colitis. This protection is achieved by inhibiting IL-13 production by ILC2s and NKT cells (60). After helminth infection, tuft cell-derived IL-25 constitutively activates ILC2s to produce IL-13, followed by the differentiation of tuft and goblet cells (61). Moreover, *Tritrichomonas*-generated succinate triggers tuft cell hyperplasia via induction of the tuft cell-IL-25-ILC2-IL-13 axis (62). Lucas et al. demonstrated that IL-25 production from thymic tuft cells skews iNKT cells towards the iNKT2 phenotype in the thymus (63). Moreover, in addition to their interactions within the thymus, the interaction among tuft cells, ILC2s, and iNKT2 cells in the peripheral tissues, particularly the intestine, requires clarification.

iNKT cells (particularly IL-9-producing iNKT cells) also up-regulate IL-22-producing ILC3s in the mLNs, ultimately preventing DSS-induced colitis under IFN-γ-dysregulated conditions (23), indicating that iNKT cells and ILC3s counter-regulate IFN-γ-mediated intestinal inflammation. Moreover, IL-22 increases intestinal barrier function and anti-microbial peptide production (64, 65), and iNKT cells trigger ILC3s to produce IL-22 in the intestine (18), indicating that secretion of IL-22 by ILC3s following their interaction with iNKT cells might be effective in preventing colitis development.

### 5.3 Adaptive immune cells: CD8^+^ NKT-like cells and Tregs

A previous study demonstrated that HFD feeding increases susceptibility of mice to DSS-induced colitis via a concurrent increase in CD1d-unrestricted NKT cells (mostly CD8^+^ and DN T cells) and a decrease in Tregs (66). The severity of intestinal inflammation in transgenic mice expressing human IL-15 in IECs closely correlates with increased numbers of LP CD8^+^ NKT cells and increased levels of Th1-type cytokines such as IFN-γ and TNF-α (67). We have previously reported that CD1d-independent CD8^+^ NKT cells are critical effectors that exacerbate DSS-induced colitis in Yeti mice characterized by enhanced stability of IFN-γ mRNA transcripts. In contrast, through cooperation with Tregs, iNKT cells are required to control CD1d-independent CD8^+^ NKT cell-mediated pathogenesis during DSS-induced colitis (68). Pro-inflammatory DCs induce the differentiation of pathogenic Foxp3^−^CD25^+^CD4^+^ T cells with Th1 and Th17 phenotypes and antagonize Treg differentiation in the mLNs of CD1d^−/−^ Yeti mice lacking iNKT cells (69). iNKT cell-primed Tregs produce IL-10 in the presence of bacterial diacylglycerols and show an enhanced suppressive capacity, which provides support for the iNKT-Treg axis in regulating colitis (70). However, the regulatory effects of iNKT cells are restricted to the intestine but not to the spleen during DSS-induced colitis in Yeti mice (68), consistent with the dominance of iNKT2 cells in the intestine.

## 6 Impact of microbiota, microbiota-derived SCFA, and dietary LCFA on iNKT cells in IBD

Short-chain fatty acid (SCFA)-producing bacteria (e.g., *Faecalibacterium*, *Lachnospiraceae*, *Veillonella Gemmiger*, and *Prevotella*) help to maintain IL-10 production by iNKT cells (13). However, a previous study demonstrated that *Acetatifactor muris* (*A. muris*) belonging to *Firmicutes* is exclusively detected in the fecal samples of NOD2^−/−^CD1d^−/−^ mice lacking iNKT cells, compared to NOD2^−/−^ mice with altered intestinal microbiota. Moreover, oral gavage of *A. muris* promotes colitis in DSS-treated WT mice (71). In addition, increased colonization of pathogenic bacteria (e.g., *Pseudomonas aeruginosa* and *Staphylococcus aureus*) in the intestine of CD1d^−/−^ mice has been implicated in reduced release of Paneth cell-derived lysozyme, a potent anti-microbial protein (72). These anti-bacterial effects in WT mice are enhanced following *in vivo* iNKT cell activation by α-GalCer (72).

In DSS-induced colitis, WT B6 mice that received B6 CD1d KO-derived cecal contents show increased sensitivity to colitis compared with control WT B6 mice injected orally with WT B6-derived cecal contents. Furthermore, an increase in particular segmented filamentous bacteria and a decrease in *Akkermansia muciniphila* (*A. muciniphila*) closely correlates with increased colitis sensitivity in WT B6 mice that received B6 CD1d KO-derived cecal contents (73). Although oral administration of *A. muciniphila* plays a protective role in the Oxa-induced colitis model and *A. muciniphila* stimulates macrophages to release IL-10 (74), it remains unclear whether IL-10 production by *Akkermansia*-stimulated macrophages can induce iNKT10 differentiation in the colon during UC. Thus, it will be important to perform further studies on the protective mechanism of *Akkermansia* in colitis.

The kynurenic acid-GPR35 signaling pathway ameliorates DSS-induced colonic injury and inflammation by suppressing NLRP3-dependent IL-1β production in macrophages (75). Peripheral iNKT cells express high surface levels of GPR35, and the specific activation of GPR35 by its ligand (kynurenic acid) significantly reduces the release of IL-4 but not IFN-γ by α-GalCer-activated human iNKT cells (76). In addition, GPR65 gene expression in intestinal tissues is significantly up-regulated in both CD and UC patients compared with healthy controls (77). Moreover, the progression of DSS-induced colitis is aggravated by GPR65 deficiency, which supports the protective role of GPR65 in this model (77). iNKT cells expressing high levels of GPR65 play a pivotal role in suppressing autoimmune disease. For example, GPR65-deficient mice are more sensitive to experimental autoimmune encephalomyelitis in an iNKT cell-dependent manner (78). Collectively, these studies suggest that G protein-coupled receptors such as GPR35 and GPR65 play essential roles in IBD protection through iNKT cells.

Butyrate, one of the SCFAs produced by microbiota, suppresses iNKT cell production of both IFN-γ and IL-4 via inhibiting class I histone deacetylases (79). In addition, palmitic acid (C16:0), a saturated fatty acid found in animal and vegetable fats, reduces the levels of both IFN-γ and IL-4 via inositol-requiring enzyme 1α (IRE1α) (80). Moreover, palmitic acid directly triggers NK1.1-negative iNKT cells to produce anti-inflammatory cytokines (e.g., IL-10) via IRE1α-X-box binding protein 1 (81). Thus, combining butyrate and palmitic acid as IFN-γ/IL-4 dual antagonists might represent a promising strategy to induce iNKT cell-mediated suppression in Th1 cell-mediated CD and Th2 cell-mediated UC.

## 7 Concluding remarks

**Figure 1:**
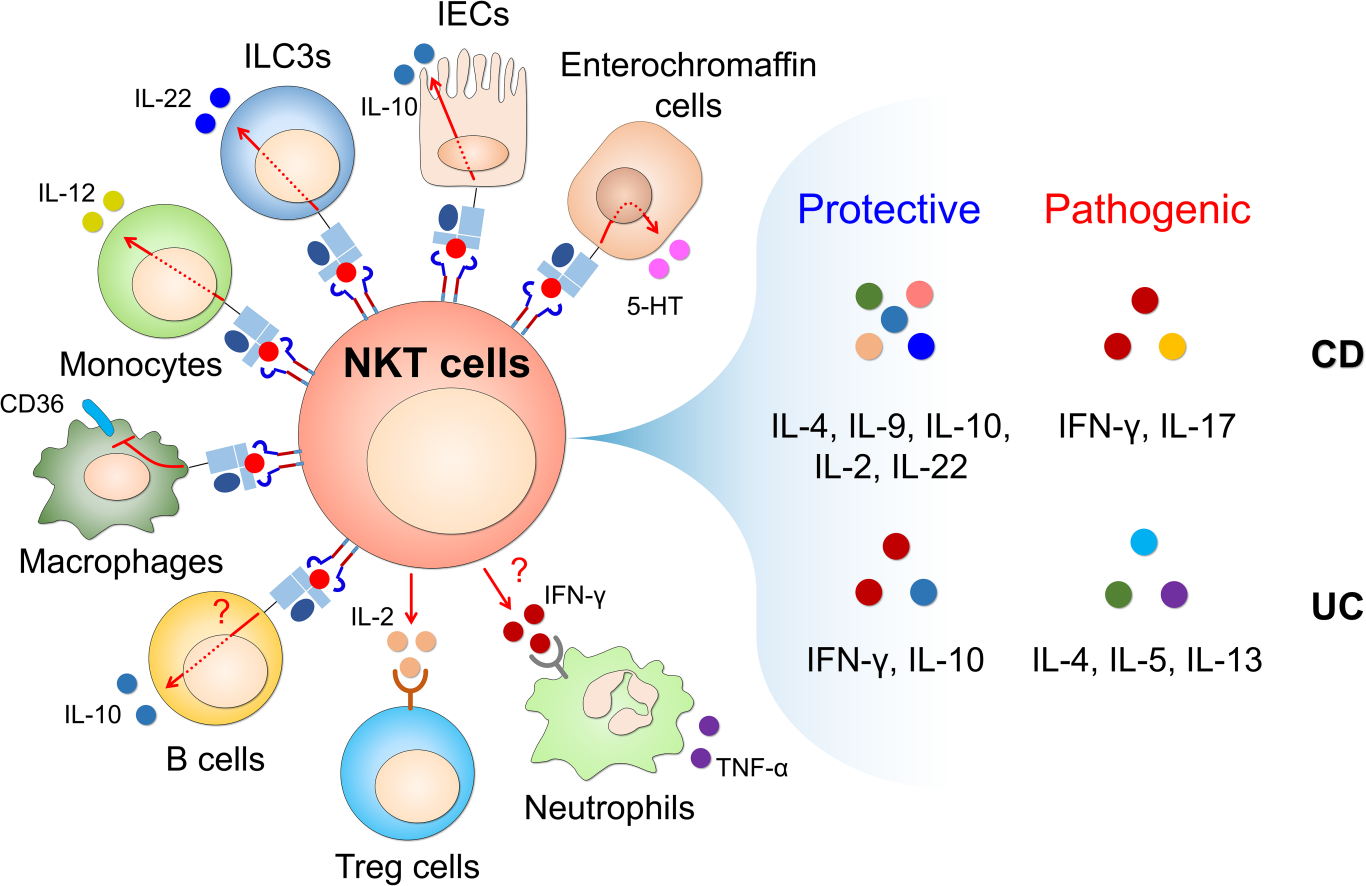
CD1d-restricted NKT cells interact with CD1d-expressing cells to produce NKT cell-derived soluble factors that regulate intestinal inflammatory responses. Glycolipid-mediated CD1d engagement triggers the activation of CD1d-expressing non-immune cells (such as IECs and enterochromaffin cells) and immune cells (such as ILC3s, B cells, monocytes and macrophages). This activation occurs through CD1d-intrinsic signaling in non-immune and immune cells and TCR/cytokine-dependent mechanisms in NKT cells. In addition, NKT cells interact with Tregs and neutrophils in a cytokine-dependent manner. Through this cross-talk with diverse cell types, CD1d-restricted NKT cells rapidly produce T helper (Th)1 cytokines (e.g., IFN-γ), Th2 cytokines (e.g., IL-4, IL-5, IL-9, and IL-13), Th17 cytokines (e.g., IL-17 and IL-22), and regulatory cytokines (e.g., IL-10) upon stimulation with various stimuli (e.g., cytokines, chemokines, toll-like receptor (TLR) ligands, fatty acids, and glycolipids), which can elicit either protective or pathogenic effects in CD and UC. CD, Crohn’s disease; UC, ulcerative colitis; IECs, intestinal epithelial cells; ILC3s, type 3 innate lymphoid cells; 5-HT, 5-hydroxytryptamine.

The studies reviewed here identify iNKT cells as promising targets for designing IBD immune therapies (**Figure 1** and **Table 1**). Although emerging evidence shows the immunological functions of CD1d and iNKT cell subsets (iNKT1, iNKT2, iNKT17, and iNKT10), little is known about their contribution to protection/pathogenesis against CD and UC. Further investigations are needed to explore the precise immunoregulatory mechanisms of intestinal iNKT subsets during IBD. Finally, it will be important to develop new tools to selectively activate or inhibit iNKT cells in the intestine.

**Table 1:** Roles of gut microbiota and their derivatives in iNKT cell immunity.

Gut microbiota	Glycolipid Ag /TLR ligands/others	CD1d dependency	TLR dependency	Functions	References
*Bacteroides fragilis*	α-GalCer-like (d17-19:0;βh17:0)	dependent	–	Induce IFN-γ and IL-2 production by iNKT cells	(82)
	polysaccharide A	–	dependent (TLR2)	Induce thymic PLZF^+^ iNKT cell development	(83)
	*GSL* **-** *Bf717*	dependent	–	Inhibit EC-derived serotonin release induced by iNKT cell-mediated CD1d ligation	(16)
	*B. fragilis* α-GalCer	dependent	–	Induce activation of and IFN-γ production by iNKT cells	(84)
	*GSL* **-** *Bf717*	dependent	–	Inhibit iNKT cell activation and iNKT cell-mediated colitis	(41)
*E. coli LF82*	–	dependent	–	Induce IFN-γ production by iNKT cells	(30)
*Salmonella typhimurium*	–	dependent	–	Induce IFN-γ production by iNKT cells	(30)
*Sphingobium* *yanoikuyae*	–	dependent	–	Induce iNKT cell activation	(32)
*Helicobacter* *pylori*	Cholesteryl α-Glucosides	dependent	–	Induce iNKT cell activation	(85)
*Clostridium* *scindens*	bile acid	–	–	Induce a rapid reduction of hepatic iNKT cells	(86)

–, not evaluated; EC cells, enterochromaffin cells.

## Author contributions

SL: Funding acquisition, Writing – original draft, Writing – review & editing. HP: Funding acquisition, Writing – original draft, Writing – review & editing. LVK: Writing – review & editing. SH: Funding acquisition, Supervision, Writing – original draft, Writing – review & editing.
